# Robust Detection of Rare Species Using Environmental DNA: The Importance of Primer Specificity

**DOI:** 10.1371/journal.pone.0059520

**Published:** 2013-03-26

**Authors:** Taylor M. Wilcox, Kevin S. McKelvey, Michael K. Young, Stephen F. Jane, Winsor H. Lowe, Andrew R. Whiteley, Michael K. Schwartz

**Affiliations:** 1 United States Department of Agriculture Forest Service, Rocky Mountain Research Station, Missoula, Montana, United States of America; 2 Department of Environmental Conservation, University of Massachusetts, Amherst, Massachusetts, United States of America; 3 Division of Biological Sciences, University of Montana, Missoula, Montana, United States of America; University of Houston, United States of America

## Abstract

Environmental DNA (eDNA) is being rapidly adopted as a tool to detect rare animals. Quantitative PCR (qPCR) using probe-based chemistries may represent a particularly powerful tool because of the method’s sensitivity, specificity, and potential to quantify target DNA. However, there has been little work understanding the performance of these assays in the presence of closely related, sympatric taxa. If related species cause any cross-amplification or interference, false positives and negatives may be generated. These errors can be disastrous if false positives lead to overestimate the abundance of an endangered species or if false negatives prevent detection of an invasive species. In this study we test factors that influence the specificity and sensitivity of TaqMan MGB assays using co-occurring, closely related brook trout (*Salvelinus fontinalis*) and bull trout (*S. confluentus*) as a case study. We found qPCR to be substantially more sensitive than traditional PCR, with a high probability of detection at concentrations as low as 0.5 target copies/µl. We also found that number and placement of base pair mismatches between the Taqman MGB assay and non-target templates was important to target specificity, and that specificity was most influenced by base pair mismatches in the primers, rather than in the probe. We found that insufficient specificity can result in both false positive and false negative results, particularly in the presence of abundant related species. Our results highlight the utility of qPCR as a highly sensitive eDNA tool, and underscore the importance of careful assay design.

## Introduction

Environmental DNA (eDNA) is DNA extracted from an environmental sample without isolating the target organism [1], [2]. Environmental DNA has been characterized as a mixture of genomic DNA from many different organisms, which is often degraded into small fragments [2]. The collection and analysis of eDNA has many applications; one that has recently received a great deal of attention is the detection of rare aquatic vertebrates, both for the early detection of invading nonnative species (e.g., [3]–[6]) and for the detection of rare native species of interest (e.g., [7]–[9]) such as species currently listed under the U.S. Endangered Species Act.

Much has been written about the problems associated with nonnative species, from economic to ecological impacts (e.g., [10], [11]). Nonnative species pose a particular threat to closely related native species with which they may compete or hybridize (e.g., [12], [13]). Consequently, they, and the closely related native species that they impact, are often the target of extensive monitoring programs. Environmental DNA can be a useful tool during three periods of nonnative species invasions: (1) early in the invasion when invaders are rare, (2) late in the invasion when native species are rare, and (3) following management control actions designed to eradicate the invading species. Environmental DNA monitoring could be a useful approach in these situations because conventional survey methods require inordinately large amounts of effort to achieve adequate levels of detection when species are rare [5].

Along with its unique advantages, eDNA monitoring presents a unique set of challenges and limitations. For example, typical non-invasive DNA sampling targets a particular species by sampling hair, scat or other tissues deposited by the target organism. Thus, most of the DNA will either be from the target species, or from distantly related species (e.g., bacteria or prey species). In eDNA approaches, target organism DNA likely represents a minority of the total DNA collected, and the sample may be dominated by DNA from closely related, non-target species. Although eDNA from aquatic systems can be concentrated via precipitation [4] or filtration [7], there is no way to increase the relative proportion of target DNA in the sample prior to PCR amplification.

Stream systems also pose special challenges for eDNA analyses. In lentic systems (e.g., ponds and lakes), equilibrium DNA concentrations are determined primarily by the rate of new DNA entering the water and the rate of DNA leaving the system through degradation [8], [14]. In lotic systems (e.g., streams and rivers), downstream transport likely causes DNA concentrations to be much lower than in lentic systems [8], especially if the target species is rare. For eDNA to be useful as a monitoring tool for rare species in moving water we need to ascertain the sensitivity of an assay and relate this to organism abundance and stream discharge (and hence to DNA quantity per liter). If the target organism co-occurs with closely related species, we need to determine the assay’s reliability when the target DNA represents a fraction of closely related non-target DNA present.

Quantitative PCR (qPCR) using TaqMan™ assays with minor groove binding probes (TaqMan MGB; Applied Biosystems – Life Technologies Corporation) may be a particularly useful tool for eDNA. TaqMan MGB assays use two primers to amplify a small section of target template. Nested within this amplicon is a binding site for a probe, which is labeled with a fluorescent reporter dye on one end and a quencher molecule on the other. When intact, the quencher absorbs the reporter dye signal via fluorescent resonance energy transfer (FRET). The probe is cleaved by the 5′–3′ nuclease activity of the DNA polymerase during extension, thus separating the quencher and resulting in an increased fluorescent signal. As a result, the level of fluorescence is proportional to the quantity of template. As PCR progresses and quantity of amplicon increases, so does the level of fluorescence, creating an amplification curve where fluorescence increases rapidly early in PCR, but slows to a plateau as reagents are consumed. The greater the initial quantity of target template, the earlier in PCR this curve becomes detectable. The cycle number at which the curve becomes clearly distinguishable from the background fluorescence is called the C_t,_ and it can be compared to standards of known copy number to accurately estimate initial target quantity in the unknown sample [15]. This method is known to be highly sensitive. For example, previous research used repeated dilutions to assess the sensitivity of traditional PCR tests for eDNA detection of Asian carp (*Hypophthalmichthys molitrix* and *H. nobilis*) [5], [16]. The researchers determined that these species could be detected at concentrations of 7 copies/µL of DNA for *H. molitrix* and 207 copies/µL for *H. nobilis* using 1 µL template in traditional PCR reactions [5], [16]. In comparison, qPCR has been shown to have reasonable probabilities of detection at concentrations as low as 0.5 copy/µL [17].

TaqMan MGB probes should also be substantially more target-specific than traditional PCR. Minor groove binding probes use a 3′ modification to allow construction of very short probes and are extremely sensitive to base pair mismatches [18]. These types of assays have been used for several recent eDNA studies [8], [19]–[21]. However, even TaqMan MGB probes can produce measureable signals with two or three base pair mismatches [22]–[24]. Location of the base pair mismatches within the probe [18] and the primer sets [25] are important to assay specificity. Thus, when using TaqMan MGB assays, accurate results rely on careful assay design, especially when the target species represents a small proportion of the total sample DNA and a closely related species represents the majority.

In the western United States, brook trout (*Salvelinus fontinalis*) are an invasive fish species that has been widely stocked into small streams, where it commonly displaces native salmonids including bull trout (*Salvelinus confluentus*) (e.g., [26]) and cutthroat trout (*Oncorhynchus clarkii*) (e.g., [27]). To create sanctuaries for native fishes, fish migration barriers are often constructed, followed by brook trout removal above these barriers [28]. Complete eradication of brook trout is essential for this approach to be effective, and frequent monitoring is required to confirm barrier efficacy [28]. Thus, detection of brook trout is critical at the initial stage of an invasion when suppression may be most effective, as well as when evaluating removal efforts and barrier effectiveness. Bull trout are of concern because they are currently listed as Threatened in the United States under the U.S. Endangered Species Act [29]. This management status places legal restrictions in waters where bull trout occur, yet this species frequently exists at low densities and is difficult to detect using conventional methods [30]. Thus, both species are good candidates for eDNA-based sampling, assuming that eDNA can detect their presence at extremely low densities. Furthermore, because they co-occur, it is important to be able to detect rare bull trout when brook trout are common and vice-versa.

Here we develop TaqMan MGB assays for detection of these two closely related species of char. We focus on testing the reliability and sensitivity of an assay for detection of brook trout––both in detecting isolated brook trout DNA and when applied to mixtures of DNA from multiple species.

## Materials and Methods

### Assay Design

We designed two TaqMan MGB assays for detection of brook trout and one assay for detection of bull trout. These assays target ∼150 base pair sites on the *cytochrome b* gene (*cyt b*) which are highly divergent across species of salmonids that often co-occur in the western United States [31]. We targeted mitochondrial markers because there are substantially more mitochondrial DNA (mtDNA) copies per cell than nuclear DNA copies; which is why mtDNA is commonly targeted when DNA is present at low concentrations and/or degraded [32].

One brook trout assay (*BRK1*) was designed to maximize base pair differences between brook trout and non-brook trout salmonids within the *probe*-binding region (Table 1). Base pair mismatches in the probe-binding region reduce the affinity of the probe for the template and are intended to cause reduced or no fluorescence of non-targets, even if primers bind and there is resulting amplification. The second brook trout assay (*BRK2*) and bull trout assay (*BUT1*) were designed to maximize the base pair differences between target (brook trout and bull trout, respectively) and non-target salmonids in the *primer*-binding regions and contain at least one base-pair difference in the probe-binding region (Table 1). Base pair mismatches in the primer-binding regions reduce affinity of the primers for the template and are intended to cause reduced or no amplification of non-targets.

**Table 1:** Assay sequences and measures of specificity.

Assay	Non-target	F primer mismatches (5′–3′)	R primer mismatches (5'–3)	Probe mismatches (5′–3′)	Proportional S/N
BRK1	Bull trout	CCATGAGGGCAAATATCCTTCTGA	TCATTGTACAAGGGCACCTCCTA	CTCCTCTCTGCTGTACCC	0.56 (0.003)
	Lake trout	CCATGAGGGCAAATATCCTTCTGA	TCATTGTACAAGGGCACCTCCTA	CTCCTCTCTGCTGTACCC	0.30 (0.002)
	Brown trout	CCATGAGGGCAAATATCCTTCTGA	TCATTGTACAAGGGCACCTCCTA	CTCCTCTCTGCTGTACCC	0.28 (0.002)
BRK2	Bull trout	CCACAGTGCTTCACCTTCTATTTCTA	GCCAAGTAATATAGCTACAAAACCTAATAGATC	ACTCCGACGCTGACAA	0.18 (0.003)
	Lake trout	CCACAGTGCTTCACCTTCTATTTCTA	GCCAAGTAATATAGCTACAAAACCTAATAGATC	ACTCCGACGCTGACAA	0.18 (0.001)
	Brown trout	CCACAGTGCTTCACCTTCTATTTCTA	GCCAAGTAATATAGCTACAAAACCTAATAGATC	ACTCCGACGCTGACAA	0.18 (0.001)
BUT1	Brook trout	AGTACTTCACCTTCTGTTTCTGCATG	C AATATAGCTACGAAACCGAGGAGG	CCGACAAAATCTCA	0.27 (0.009)
	Lake trout	A GTACTTCACCTTCTGTTTCTGCATG	C AATATAGCTACGAAACCGAGGAGG	CCGACAAAATCTCA	0.28 (0.005)
	Brown trout	AGTACTTCACCTTCTGTTTCTGCATG	C AATATAGCTACGAAACCGAGGAGG	CCGACAAAATCTCA	0.21 (0.026)

Primer and probe sequences and location of mismatches (underlined) with non-target taxa for the three assays. Proportional S/N is the normalized fluorescence (Rn) at 40 cycles divided by the Rn at 1 cycle, standardized by the S/N of a positive control (n = 3; mean and std).

Because no published *cyt b* sequence data were available for bull trout when developing these assays, we used primers developed for brook trout (unpublished data), Arctic char (*Salvelinus alpinus*) [33] and sculpin (*Cottus spp.*) [34], [35] to sequence a 708, 838, and 800 bp region of *cyt b* for 10 bull trout, 6 brook trout and 2 lake trout (*Salvelinus namaycush*) respectively. Brook trout and bull trout were collected from 10 and 6 streams respectively, distributed across western Montana and northern Idaho (Montana Fish, Wildlife and Parks Scientific Collectors Permits 12–2001, 14–2010, and 19a-2009, U.S. Fish and Wildlife Service Federal Fish and Wildlife Permit TE220826-0). Lake trout were collected from Flathead Lake, Montana by The Confederated Salish and Kootenai Tribes, Division of Fish, Wildlife, Recreation, and Conservation. All brook trout were derived from introduced populations, but the lineages are of unknown origin. Lake trout were stocked into Flathead Lake in the early 20^th^ century from an unknown source (C.P. Stafford, personal communication). DNA from caudal fin tissue was extracted using the DNeasy Tissue and Blood Kit (Qiagen, Inc.) according to the manufacturer’s specifications. The bull trout and brook trout *cyt b* region was amplified using primers *Verspoor_F1* [33] and *Jane_R* (5′-CACAACTATGAGGACAAGGATCG-3′), while the two lake trout were amplified using the primers *L14724* and *H15915* [34]. Reaction volumes of 40 µl contained 50–100 ng DNA, 1× reaction buffer, 2.5 mM MgCl2, 200 µM each dNTP, 1 µM each primer, 1 U Taq polymerase (Applied Biosystems). The PCR program was 95°C/10 min, [94°C/1 min, 53°C/1 min, 72°C/1 min 30 s] × 34 cycles, 72°C/5 min. The quality and quantity of template DNA were determined by 1.8% agarose gel electrophoresis and a 1 kb ladder. PCR products were purified using ExoSap-IT (Affymetrix-USB Corporation) according to manufacturer’s instructions. DNA sequence data was obtained using the Big Dye kit and a 3700 DNA Analyzer (ABI; High Throughput Genomics Unit, Seattle, WA, USA) using the primers *Verspoor_F1, Verspoor_F2,* [34] *Jane_F* (5′-GATTAACTCCGACGCTGACAA-3′) and *Jane_R* (above) for bull trout and brook trout, and the primers *Lc2, Lc3* [35] and *H15915* [34] for lake trout. All primers were ordered from Integrated DNA Technologies (IDT). These sequence data are available on GenBank (accession numbers KC344819–KC344826).

We used published brook trout sequence data (Genbank, accession number AF154850.1) to select a probe-binding region for the *BRK1* assay. This region was compared with published sequences for closely related species, including Dolly Varden (*Salvelinus malma*), lake trout, and Arctic char (*Salvelinus alpinus*) (GenBank accession numbers JN868488.1, DQ451389.1, and NC_000861.1) to maximize within-probe base pair mismatches. Primers for a region surrounding this probe were then designed by the assay manufacturer (Applied Biosystems) to optimize assay performance. To design the *BRK2* and the *BUT1* assays, we used sequences generated for brook trout, bull trout, and lake trout (above), as well as published sequences including brook trout, brown trout (*Salmo trutta*), Arctic grayling (*Thymallus arcticus*), and rainbow trout (*Oncorhynchus mykiss*) (GenBank accession numbers AF154850.1, NC_010007, NC_012929.1, and NC_001717.1) to maximize within-primer base pair mismatches. These assays were designed using PrimerExpress v3.0 software (Applied Biosystems). One bull trout that was sequenced was found to differ at a single base pair within the forward primer region of the *BUT1,* however, this did not show any effect on assay efficiency.

All assays were obtained from Applied Biosystems, and contained a primer set and a FAM-labeled minor groove binding, non-fluorescent quencher (MGB-NFQ) probe (Table 1). All experiments were run in 20 µl volumes with 4 µl of template, 10 µl TaqMan Fast Universal Master Mix (Applied Biosystems), 2 µl assay (primers each at 18 µM, probe at 5 µM), and 4 µl diH_2_O following fast cycling conditions (95°C/20 s [95°C/1 s, 60°C/20 s]×45 cycles) on a StepOne Real-time PCR Instrument (Applied Biosystems). Because real-time PCR is highly sensitive, and therefore susceptible to contamination, all experiments were set up inside of an enclosure which was irradiated with UV for 1 h prior to each use, along with all consumables and pipettes. Reagents were also aliquoted into small quantities prior to experiments such that each reagent tube was only opened a single time in a PCR product-free environment.

### Field Collection

We collected eDNA from two streams in west-central Montana known to contain brook trout: Plant Creek, (46° 43′ 19″, 113° 54′ 22″) which is dominated by brook trout and does not contain any other char species (M.K. Young, unpublished data; Montana Fish, Wildlife and Parks MFISH database, http://fwp.mt.gov/fishing/mFish/), and Miller Creek (46° 45′ 8″, 113° 56′ 38″), which contains a mixture of brook trout and non-target salmonids (Montana Fish, Wildlife and Parks MFISH database, http://fwp.mt.gov/fishing/mFish/). Similar to some previous studies [5], [7], samples were collected by pumping 6 L of water through a Whatman 47-mm diameter, 1.5-µm glass microfiber filter (GE HealthCare) using a series II Geopump™ peristaltic pump (Geotech Environmental Equipment Inc.). Filters were stored on ice until arrival at the lab. Within 24 h following arrival, genomic DNA was extracted from all filters using the MoBio Powerwater™ DNA Extraction Kit (MoBio Laboratories, Inc.) following the manufacturer protocol, except that the final elution was done with 100 µl of IDTE pH 8.0 (10 mM Tris pH 8.0, 0.1 mM EDTA; Integrated DNA Technologies) in place of the manufacturer-provided elution buffer. The resulting extracted DNA and buffer was stored at −20°C until further use. Tubing and filter holders were sterilized with a 10% chlorine bleach solution between samples.

### Assay Sensitivity

We determined assay sensitivity by repeatedly testing amplification success across a set of serial DNA dilutions. We used four different stock solutions: two stock solutions were from tissue samples, one contained exclusively brook trout DNA, and one contained 10% brook trout and 90% bull trout DNA. In addition, we diluted two samples of eDNA, one from Plant Creek and one from Miller Creek (above). The brook trout DNA solution was created by digesting brook trout tissue in 180 µl ATL (tissue lysis buffer; Qiagen) and 20 µl ProteinaseK (Qiagen), then pouring this solution onto a paper filter which was then extracted using the MoBio Powerwater™ DNA Extraction Kit (above). DNA extracted for sequencing (above) was used for the brook/bull trout mixed solution. These two tissue-derived solutions were diluted 1∶100 in IDTE pH 8.0 prior to quantification.

Quantification and probability of detection experiments were all performed using the BRK2 assay. To quantify the initial copy numbers of the four stock solutions of DNA prior to dilution, we used a synthetic gene from Integrated DNA Technologies (IDT) containing the 139-bp sequence of interest (probe and primers) to create a standard curve. The synthetic gene arrived desiccated and was re-suspended in 200 µl of IDTE pH 8.0. We then linearized 20 µl of this stock using Pvu1 restriction digest (New England BioLabs) and purified this product using the PureLink PCR Micro Kit (Invitrogen – Life Technologies Corporation). The final elution using the PureLink elution buffer was 10 µl, to which we added 10 µl of IDTE pH 8.0 for a final volume of 20 µl. This solution was quantified on a Qubit 2.0 fluorometer (Invitrogen), and diluted in IDTE pH 8.0 to a final stock concentration of 10^6^ copies/µl. This solution was then serially diluted to create known quantity standards to estimate brook trout mtDNA concentration in the four sources of DNA over a series of two PCR plates. Each standard, unknown quantity sample, and a no template control (NTC) were run in triplicate on each plate. We used these qPCR quantification data to estimate brook trout mtDNA copy number concentration for each of the four stock solutions (Table 2). Based on these quantifications, the tissue-extracted brook trout sample was diluted into IDTE pH 8.0 to estimated concentrations of 312.5, 62.5, 12.5, 2.5, and 0.5 copies/µl; the other three samples were diluted into IDTE pH 8.0 to estimated concentrations of 12.5, 2.5, and 0.5 copies/µl.

**Table 2:** Quantification of stock solutions for assay sensitivity experiments.

Estimated copies of brook trout DNA per µl								
Replicate					Standard curve summary			
	1	2	3	Average	std	r^2^	Efficiency (%)	Y-intercept
Brook trout tissue	1206.3	1204.3	1172.8	1194.4	18.79	0.999	92.24	40.13
Plant Creek	469.8	497.3	523.8	496.9	27.00	0.999	93.49	40.51
Miller Creek	104.5	110.0	101.5	105.3	4.31	0.999	93.49	40.51
Brk:But 1∶10 mixture	606.3	581.5	595.3	594.3	12.40	0.999	93.49	40.51

Estimated copy number of samples prior to dilution and comparisons to the standard curve. R^2^>0.99 and efficiencies 90–110% are necessary for accurate target quantification (Agilent Technologies Methods and Applications Guide).

We developed a probability of detection curve from each dilution series by running multiple replicates of each dilution. The brook trout tissue-extracted sample dilution series was run a total of 40 replicates per dilution. The Plant Creek, Miller Creek, and the brook trout-bull trout mixed DNA dilutions were run a total of 26 replicates at the 12.5 target copies/µl dilutions and 32 replicates at the 2.5 and 0.5 target copies/µl dilutions. Each PCR plate included a NTC in triplicate. A replicate was counted as a success if there was amplification above the threshold level of fluorescence within 45 cycles. The probability of detection at each dilution was calculated as the proportion of successes out of the total number of replicates.

### Assay Specificity

Specificities of the three assays were compared using DNA of target (brook trout or bull trout) and non-target salmonids (brook trout, bull trout, lake trout, and brown trout) as the PCR template. DNA was extracted from a piece of fin tissue from brook trout, bull trout, lake trout, and brown trout (Montana Fish, Wildlife and Parks Scientific Collectors Permits 12–2001, 14–2010, and 19a-2009, U.S. Fish and Wildlife Service Federal Fish and Wildlife Permit TE220826-0) using the Qiagen DNeasy® Tissue and Blood Kit (Qiagen) following the manufacturer’s instructions. Reactions for each DNA template source were run in triplicate to identify outliers. To standardize specificity measures, we measured the signal divided by the noise (S/N), which is endpoint fluorescence (we use normalized fluorescence (Rn) at 40 cycles) divided by fluorescence at a single cycle [24]. We scaled this measure, by dividing the S/N of each sample by the triplicate average S/N of a positive control (brook trout DNA for *BRK1* and *BRK2*, bull trout DNA for *BUT1*), expressed as “proportional S/N.” The target species has a proportional S/N of 1.0, and in the ideal assay, all non-targets would have a proportional S/N equal to that of a NTC (no amplification).

### Non-target Template Competition

We used extracted DNA from the assay specificity tests (above) to compare the ability of the three assays to distinguish target DNA in the presence of high concentrations of non-target DNA. For the *BRK1* and *BRK2* assays, extracted brook trout DNA (target) was diluted into bull trout and lake trout DNA (non-target) solution at ratios of 1∶10 and 1∶100. For the *BUT1* assay, extracted bull trout DNA (target) was diluted into brook trout and lake trout DNA (non-target) solution at ratios of 1∶10 and 1∶100. Each of these dilutions was run in triplicate.

## Results

### Assay Sensitivity

The standard curves were suitable for approximating copy number of unknown samples (Agilent Technologies Methods and Applications Guide; Table 2), and we had adequate copy numbers from all stock solutions to perform dilutions. We found high probabilities of detection, even at very low copy numbers. In all four dilution series there was 100% amplification success at estimated concentrations of 10 copies per reaction and greater (i.e., ≥2.5 copies/µl). At an estimated concentration of two copies per reaction (i.e., 0.5 copies/µl), probability of detection decreased, but in all cases was >0.700 (0.825, 0.719, 0.862, and 0.844 for brook trout tissue-extracted DNA, Plant Creek filter, Miller Creek filter, and brook/bull trout DNA dilution, respectively). If we consider a sampling session as a triplicate of samples, the amplification success was >0.975 (Table 3).

**Table 3:** Assay *BRK2* sensitivity results.

DNA source	DNA concentration (copies/µl)	n	Proportion successful
Brook trout tissue	312.5	40	1
	62.5	40	1
	12.5	40	1
	2.5	40	1
	0.5	40	0.825
Plant Creek	12.5	26	1
	2.5	32	1
	0.5	32	0.719
Miller Creek	12.5	26	1
	2.5	32	1
	0.5	32	0.862
Brk:But 1∶10 mixture	12.5	26	1
	2.5	32	1
	0.5	32	0.844

The DNA source (brook trout tissue, Plant Creek and Miller Creek filter extractions, and a mixed brook/bull trout DNA solution diluted 1∶10), estimated concentration, number of replicates, and proportion of replicates with amplification for the assay sensitivity experiments. All DNA sources had 100% amplification success when concentrations were ≥2.5 copies/µl, and >70% amplification success when concentrations were 0.5 copies/µl.

### Assay Specificity

**Figure 1:**
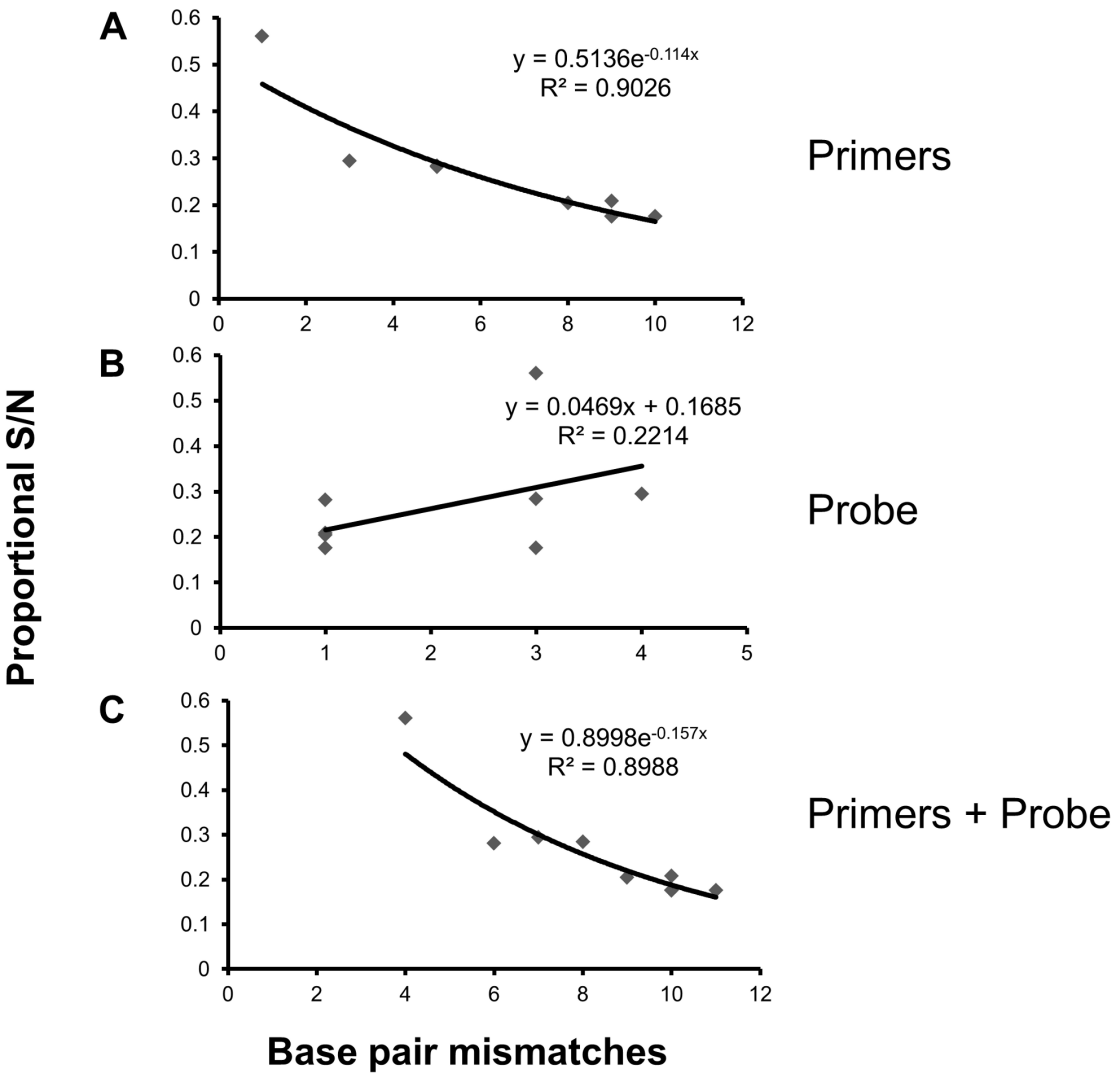
Impact of base pair mismatches on fluorescence. The relationship between end-point fluorescence (measured as proportional S/N; the fluorescence at cycle 40 divided by the fluorescence at cycle 1 as a proportion of a positive control) and base pair mismatches in the primer regions (A), probe region (B), and primers and probe regions combined (C). End-point fluorescence decreases as the number of primer base pair mismatches increases.

**Figure 2:**
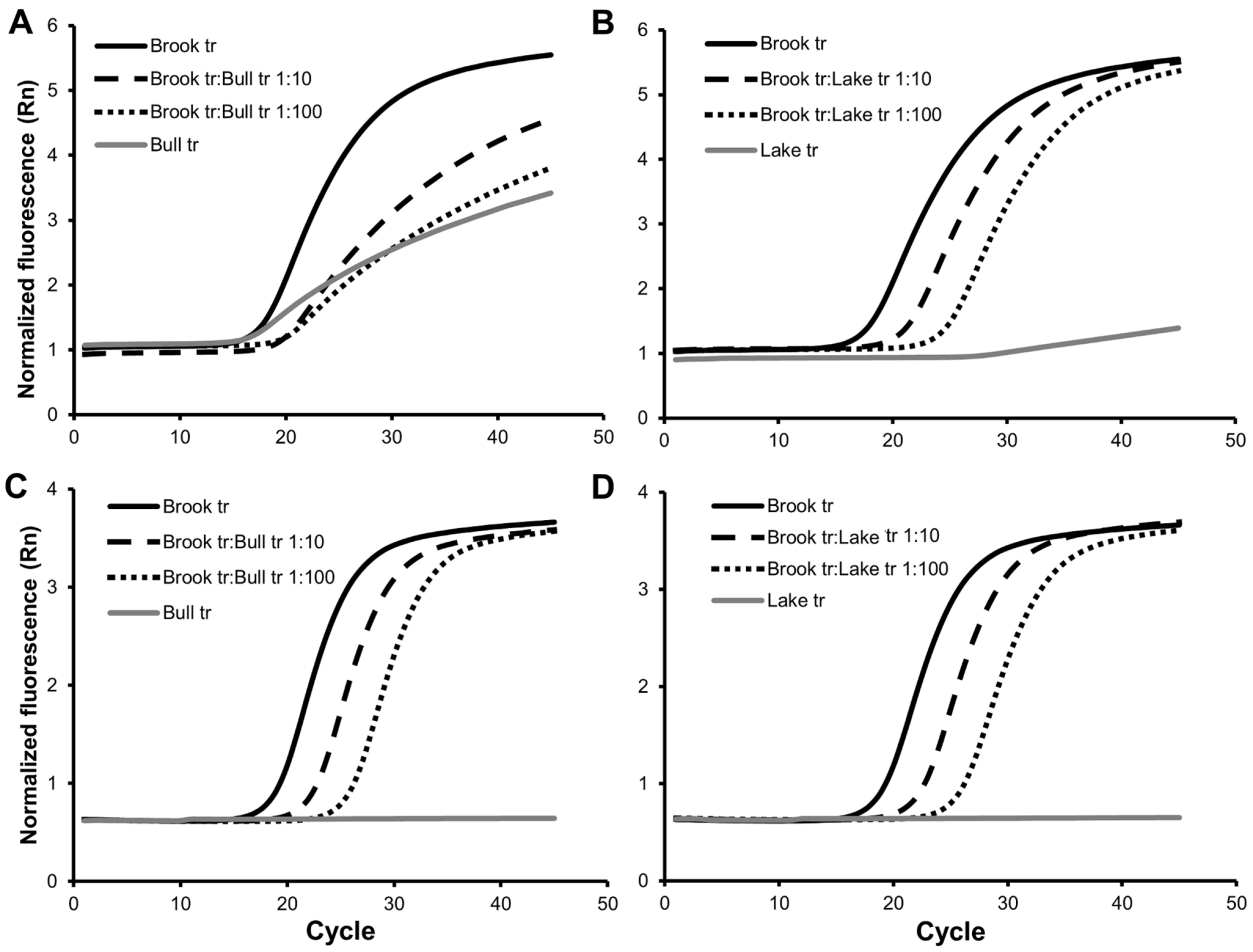
Non-target template competition in the brook trout assays. Part A shows the amplification curves of *BRK1* when using DNA solutions from brook trout, bull trout, and brook trout mixed into bull trout at 1∶10 and 1∶100 dilutions. Part B shows the amplification curves of *BRK1* when using DNA solutions from brook trout, lake trout, and brook trout mixed into lake trout at 1∶10 and 1∶100 dilutions. Part C shows the amplification curves of *BRK2* when using DNA solutions from brook trout, bull trout, and brook trout mixed into bull trout at 1∶10 and 1∶100 dilutions. Part D shows the amplification curves of *BRK2* when using DNA solutions from brook trout, lake trout, and brook trout mixed into lake trout at 1∶10 and 1∶100 dilutions. Assay *BRK1* has a single primer-base-pair mismatch and three probe-base-pair mismatches with bull trout, but produces an ambiguous signal when brook trout represent a small proportion of the sample. *BRK2* has nine primer base pair mismatches and a single probe base pair mismatch with bull trout, and is still sensitive even when brook trout represents a small proportion of the sample. The presence of lake trout DNA does not appear to influence the sensitivity of either assay.

**Figure 3:**
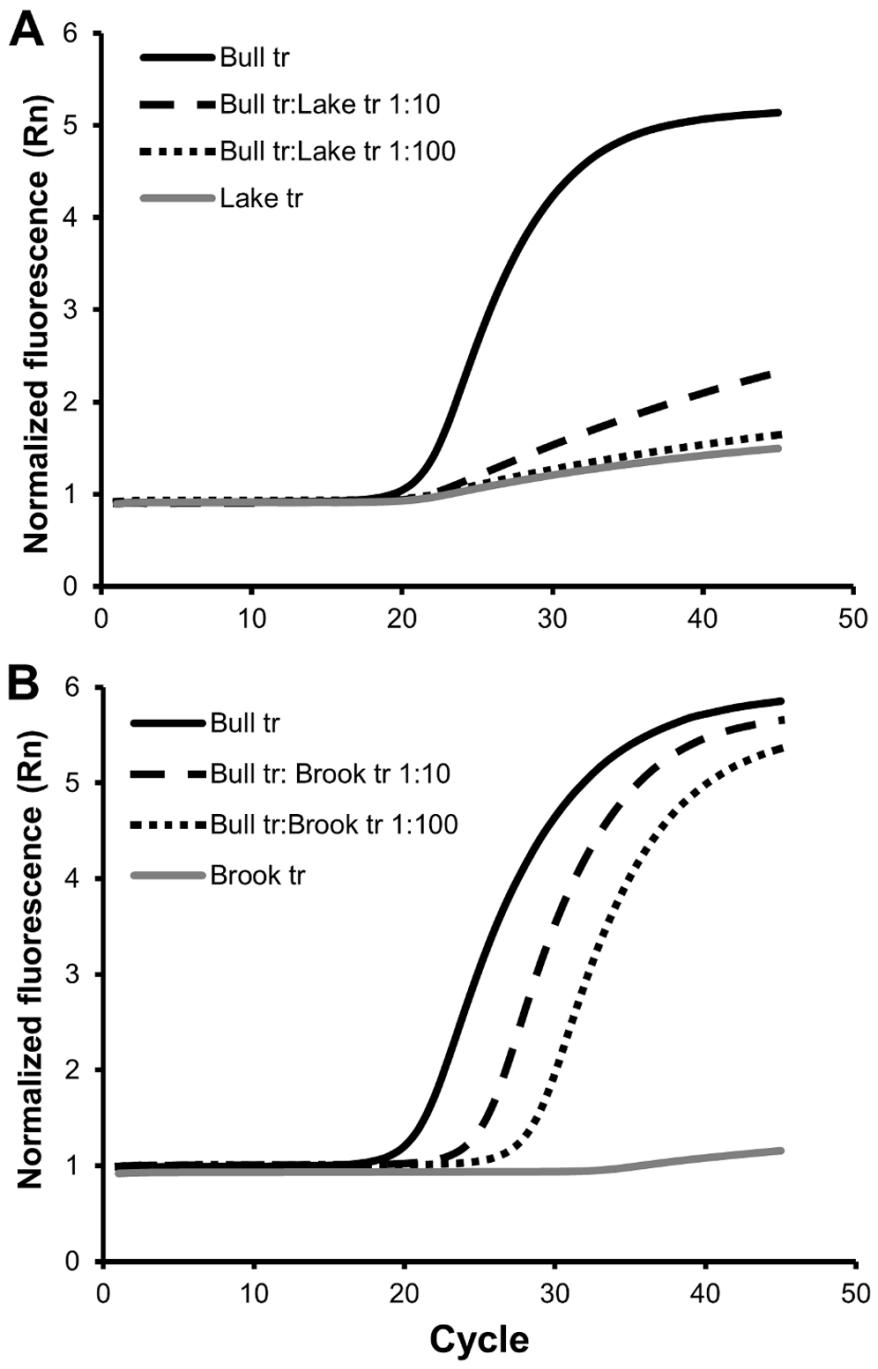
Non-target template competition in the bull trout assay. Part A shows the amplification curves of *BUT1* when using DNA solutions from bull trout, lake trout, and bull trout mixed into lake trout at 1∶10 and 1∶100 dilutions. Part B shows the amplification curves of *BUT1* when using DNA solutions from bull trout, brook trout, and bull trout mixed into brook trout at 1∶10 and 1∶100 dilutions. The assay primers have nine and five primer base pair mismatches with brook trout and lake trout respectively. Four of the mismatches with brook trout are within five base pairs of the 3′ primer ends, but none of the mismatches with lake trout are near the 3′ ends. The assay is not influenced by the presence of brook trout, but produces an ambiguous signal when bull trout represents a small portion of the sample.

In samples consisting of DNA from a single species, specificity, measured as the proportional signal to noise ratio, was correlated with the number of base pair mismatches between the assay and the species sampled (R^2^ = 0.899, Figure 1c). This was driven by base pair mismatches in the primers (R^2^ = 0.903, Figure 1a), and not by base pair mismatches in the probe-binding region, which was not significant (R^2^ = 0.221, p = 0.21, Figure 1b). In brook trout/bull trout mixed samples assay specificity was greater for *BRK2* (Figure 2c), where base pair mismatches in the primer regions were maximized between brook trout and bull trout, than for *BRK1*, where base pair mismatches in the probe region were maximized between species (Figure 2a). For *BRK1*, fluorescence degraded as the proportion of brook trout DNA in the sample decreased (Figure 2a). Additionally, the presence of bull trout DNA made sample quantification with *BRK1* inaccurate. Quantitative PCR uses the PCR cycle where the amplification curve crosses the florescence threshold (C_t_) relative to that of known standards to estimate initial template quantity; the lower the C_t_ value, the greater the estimated copy number. In the presence of bull trout DNA, the C_t_ values were less than expected, which could result in a substantial overestimate of copy number. There was no apparent effect on amplification efficiency for either brook trout assay when brook trout DNA was mixed into high concentrations of lake trout DNA solution (Figure 2b and 2d). Fluorescence of *BUT1* was unaffected when bull trout DNA was diluted into brook trout DNA solution (Figure 3b), but when bull trout DNA was diluted into lake trout DNA solution the fluorescence was degraded and the C_t_ values were strongly skewed downwards (Figure 3a).

## Discussion

When the target species (e.g., bull trout) is rare relative to sympatric, closely related species (e.g., brook trout), designing a target-specific assay is particularly important. We found that assay specificity was most influenced by base pair mismatches in the primers, rather than mismatches in the probe. We also found that the location of these mismatches was important. In general, as primer base pair mismatches increased, fluorescence of non-target templates decreased (Figure 1) and non-target template competition became weaker. When brook trout DNA made up ∼0.01 of the total in mixtures between brook and bull trout, the fluorescent signal was substantially reduced in assay *BRK1* (Figure 2a), which has three base-pair differences in the probe but only one in the primers (Table 1). This probably results from the bull trout DNA template competing for the primers, producing an amplification curve which is not consistently distinguishable from bull trout alone. In contrast, *BRK2* and *BUT1* have nine and eight base pair differences in the primers, respectively, but only one in the probe, and amplified as well in mixtures with bull and brook trout as they did in isolation (Figure 2c and 3b).

Base pair mismatch *location* in the primers also influenced our results. The *BUT1* assay was strongly affected in mixed bull/lake trout samples, even though there are more base pair mismatches between the *BUT1* assay and lake trout (5) than between *BRK1* and lake trout (3), which was unaffected by brook/lake trout mixtures. Base pair mismatches near the 3′ end of the primer have a much larger impact on specificity than those in other regions, and 5′ mismatches generally have very little effect on specificity (e.g., [25]). The *BRK1* assay has a base pair mismatch with lake trout in the last 2 base pairs of the 3′ end, while the *BUT1* assay base pair mismatches are further from the 3′ end (Table 1). Mismatch location is also important to *probe* specificity. For example, the *BRK1* assay produced some fluorescence using bull trout DNA template (Figure 2a). A contributing factor may be that one of the mismatches is located on the extreme 3′ end of the probe, which has a reduced effect on probe specificity [8]. A flattened amplification curve from TaqMan MGB probes with multiple base pair mismatches with non-target sequences have been observed in other studies (e.g., [22]–[24]).

If eDNA is to be used as a standard monitoring tool for streams, high sample portability and the ability to preserve samples in the field will be necessary. In lentic systems, detection of eDNA of target organisms from small amounts of water (e.g., 15 mL) has been successful [4], [8]. In streams, the DNA density of target organisms is likely to be lower because many streams are partially fed by groundwater, exposure to target organisms shedding DNA is brief, and photo-degradation of DNA is more likely because streams are shallow and well-mixed. Consequently, relatively large volumes of water may need to be sampled to concentrate an adequate DNA sample. *In-situ* filtering permits adequate sampling without the need to transport water samples. Furthermore, DNA trapped on a filter is easier to protect from degradation than DNA suspended in large volumes of water. We used ice to keep samples cold in the field, but other approaches exist (e.g., storage in 95% EtOH [7]). While brook trout were common in both of the streams we sampled, the number of copies obtained from these samples was several orders of magnitude greater than necessary to obtain reliable species identification. The final 100 µL DNA solution from our streams contained approximately 50,000 and 10,000 target copies for Plant Creek and Miller Creek, respectively.

Environmental DNA detection may be especially useful in situations where the target species is too rare to be identified using alternative approaches, or where alternative sampling approaches are unreasonably expensive or labor-intensive. However, these are also the circumstances where it is most important to understand the likelihood of false positives and false negatives. False negatives can occur because the quantity of target species eDNA collected falls below a detection threshold or because non-target substances interfere with the assay. Pumping large water samples and capitalizing on the extreme sensitivity of qPCR minimizes the risk of false negatives, as would setting the sampling scheme in a formal occupancy estimation framework (e.g., [36]). However, in this study we identified non-target template competition as another possible confounding factor. Insufficiently specific primers and probes can result in both false positives – particularly if we interpret any amplification as a positive result – and false negatives in the case of strong non-target template competition. We also found that non-target template competition strongly skewed C_t_ values, which produces inaccurate DNA quantity estimates and could lead to an inaccurate estimate of fish abundance (e.g., [19]). Furthermore, in the case of strong non-target template competition, sequencing PCR products will probably be an ineffective screen for assay specificity because the majority of products will be derived from the non-target template. Based on findings from this study, the best solution to these issues is (1) to design assays that maximize primer region base pair mismatches with non-target species, and (2) to experimentally test assays against pure and mixed samples of target and non-target DNA. Pre-screening eDNA assays against mixtures of target and non-target DNA is simple and relatively inexpensive, has been done in some other eDNA studies [7], and requires little additional effort; testing against non-target DNA is already standard in eDNA studies (e.g, [8]). There is a high cost associated with failing to detect an endangered or invasive species, so even when closely related taxa are not anticipated in the system, we believe that it is in best interests of future studies to carefully test assays against pure and mixed samples from common local species.

Hybridization poses an additional consideration when using these assays. Brook trout and bull trout are able to hybridize [37]. Because we are using mitochondrial markers, hybrid individuals could cause us to incorrectly conclude that our target species is present (e.g., detect brook trout when only bull trout and brook/bull trout hybrids are present), leading to an over-estimate of target species distribution. This problem should be less important when there is not extensive back-crossing (e.g., [38]), which appears to be true for brook trout and bull trout [37], [39]. As a result, we think that presence of mitochondrial DNA of brook trout or bull trout is indicative of species presence. This issue may be more substantial for species that more readily introgress, such as cutthroat trout and rainbow trout [12].

The hybridization issue illustrates the more general problem that target DNA presence may not always indicate target species presence. Besides hybridization, false positives may result from the extreme sensitivity of eDNA monitoring. Even when diluting to an average of two target copies/reaction, we had better than 70% amplification success with stream-derived DNA samples. This represents such a small quantity of template that detected DNA could derive from some source other than a live organism. Contamination by researchers in the field or lab is an obvious issue, but there are other possibilities as well. For example, the feces of piscivorous birds or mammals may contain prey DNA [40], and these animals may transport this DNA far from its point of origin. This issue has been a concern for on-going eDNA projects [41], and there have been previous suggestions to place a higher threshold on the acceptable copy number representing a positive identification [42]. Environmental DNA collection to detect rare organisms is relatively new, and we currently have no idea how much background DNA exists in the environment. Further studies to determine expected quantities of DNA in freshwater systems given a specific target organism biomass will help set these detection thresholds.
